# The rate of cellular hydrogen peroxide removal shows dependency on GSH: Mathematical insight into *in vivo* H_2_O_2_ and GPx concentrations

**DOI:** 10.1080/10715760701625075

**Published:** 2007-11-06

**Authors:** Chin F. Ng, Freya Q. Schafer, Garry R. Buettner, V. G. J. Rodgers

**Affiliations:** 1 Bioengineering Department, University of California, Riverside, CA 92521, USA; 2 Free Radical and Radiation Biology Program & ESR Facility, Radiation Oncology, The University of Iowa, Iowa City, IA 52242-1101, USA

**Keywords:** Glutathione, glutathione peroxidase, hydrogen peroxide, mathematical modelling, kinetics

## Abstract

Although its concentration is generally not known, glutathione peroxidase-1 (GPx-1) is a key enzyme in the removal of hydrogen peroxide (H_2_O_2_) in biological systems. Extrapolating from kinetic results obtained *in vitro* using dilute, homogenous buffered solutions, it is generally accepted that the rate of elimination of H_2_O_2_ *in vivo* by GPx is independent of glutathione concentration (GSH). To examine this doctrine, a mathematical analysis of a kinetic model for the removal of H_2_O_2_ by GPx was undertaken to determine how the reaction species (H_2_O_2_, GSH, and GPx-1) influence the rate of removal of H_2_O_2_. Using both the traditional kinetic rate law approximation (classical model) and the generalized kinetic expression, the results show that the rate of removal of H_2_O_2_ increases with initial GPx_r_, as expected, but is a function of both GPx_r_ and GSH when the initial GPx_r_ is less than H_2_O_2_. This simulation is supported by the biological observations of Li et al.. Using genetically altered human glioma cells in *in vitro* cell culture and in an *in vivo* tumour model, they inferred that the rate of removal of H_2_O_2_ was a direct function of GPx activity × GSH (effective GPx activity). The predicted cellular average GPx_r_ and H_2_O_2_ for their study are approximately GPx_r_ ≤ 1 μm and H_2_O_2_ ≈ 5 μm based on available rate constants and an estimation of GSH. It was also found that results from the accepted kinetic rate law approximation significantly deviated from those obtained from the more generalized model in many cases that may be of physiological importance.

## Introduction

### Redox reactions and ROS in biological systems

**Figure 1:**
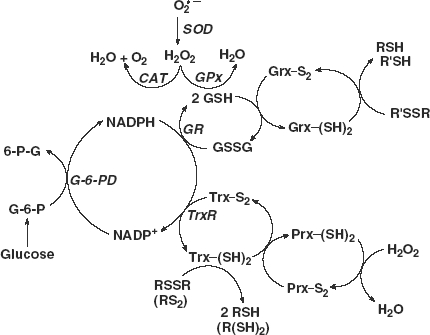
The hydrogen peroxide-removal system. There are at least three principal nodes for the removal of H_2_O_2_. Glutathione peroxidase (GPx) is a selenoenzyme that reduces H_2_O_2_ to 2H_2_O gathering the needed reducing equivalents from glutathione (GSH). The peroxiredoxin (Prx) family of enzymes is a separate node, removing H_2_O_2_ using reducing equivalents principally from thioredoxin (Trx). Catalase (CAT) is primarily located in peroxisomes; it requires no reducing cofactors to catalyse the disproportionation of H_2_O_2_.

Reactive oxygen species (ROS) (such as ${\text{O}}_{\text{2}}^{•-}$, H_2_O_2_ and organic hydroperoxides) are produced naturally in cells. They are signalling molecules, essential for the normal metabolism of cells and tissues [1–3]. High levels of ROS will lead to a more oxidized redox environment thereby inducing cell damage or even cell death [4,5]. To protect against potential oxidative damage from these species, cells and tissues have a network of antioxidant enzymes to remove these ROS (Figure 1). There are several families of enzymes that remove H_2_O_2_. This network has at least three nodes for peroxide-removal:

- Catalase is the longest known enzyme for removal of H_2_O_2_; it requires no cofactors in its catalytic mode [6];
- the six members of the peroxiredoxin family of enzymes remove H_2_O_2_ by reducing it to water and are in general recycled by gathering reducing equivalents from thioredoxin [7,8]; and
- the glutathione peroxidases rely on glutathione (GSH) for the necessary reducing equivalents.

This study focused only on the effects of GPx and GSH levels on H_2_O_2_ removal, assuming the catalase and peroxiredoxin levels were unchanged.

### GPx and GSH in removal of H_2_O_2_

In 1957 the family of glutathione peroxidases (GPx) was discovered [9]. Currently, at least four members of this family of enzymes are known [10–12]. They all reduce H_2_O_2_ to water (organic hydroperoxides are reduced to water and the corresponding alcohol) with the electrons coming from GSH, a necessary and specific cofactor.

The kinetic behaviour of GPx-1 in dilute aqueous solution is best explained by a sequence of simple bimolecular reactions [13–15]:

(1) $${\text{GPx}}_{\text{r}}+{\text{H}}_{2}{\text{O}}_{2}+{\text{H}}^{+}\overset{k_{1}}{⟶}{\text{GPx}}_{0}+{\text{H}}_{2}\text{O}$$

(2) $${\text{GPx}}_{\text{o}}+\text{GSH}\overset{k_{2}}{⟶}[\text{GS}-\text{GPx}]+{\text{H}}_{2}\text{O}$$

(3) $$[\text{GS}-\text{GPx}]+\text{GSH}\overset{k_{3}}{⟶}{\text{GPx}}_{\text{r}}+\text{GSSG}+{\text{H}}^{+}$$

yielding the overall reaction,

(4) $${\text{H}}_{2}{\text{O}}_{\text{2}}+2\text{GSH}\overset{\text{GPx}}{⟶}\text{GSSG}+2{\text{H}}_{\text{2}}\text{O}\text{.}$$

For bovine GPx-1, the kinetics of this reaction have been well studied and are considered to be a ‘ping-pong’ mechanism with indefinite Michaelis constants, indefinite maximum velocities and no significant product inhibition [10,16–22]. For this system the effective rate constants are given in Table I.

**Table I:** Rate constants for modeling the kinetic behaviour of GPx [30].

Rate constant	(m^−1^s^−1^)
*k*_1_	2.1 × 10^7^
*k*_2_	4 × 10^4^
*k*_3_	1 × 10^7^

The observations in dilute, buffered solutions lead to the paradigm that in most circumstances, the rate of peroxide removal *in vivo* is essentially independent of the concentration of GSH [16,18,23]. This assumes low levels of H_2_O_2_ (i.e. H_2_O_2_ < GPx_r_ < GSH) and, thus, the rate of recycling of GPx_r_ by GSH (equations 2 and 3) is rapid compared to the rate of the reaction of GPx_r_ with H_2_O_2_. Thus, GPx would predominantly exist in its reduced form, which is highly reactive with hydroperoxides (equation 1).

**Figure 2:**
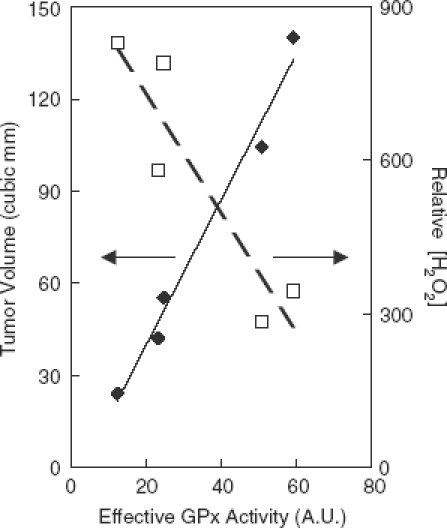
The rate of tumour growth *in vivo* varies directly with effective GPx activity (i.e. GPx × GSH); H_2_O_2_ varies inversely. (♦) Tumour volume, which is proportional to the growth rate. (□) Relative levels of intracellular H_2_O_2_ were estimated by monitoring the increase in fluorescence of 2′,7′-dichlorodihydrofluorescein (DCFH_2_). Effective GPx activity is ‘GPx-activity’ (or GPx) as measured by standard activity assay [44] multiplied by the concentration of GSH. The units are somewhat arbitrary (AU); using typical expressions of the activity of GPx (mU/mg protein) and for GSH levels (nmol/mg protein) units for effective GPx activity would be mU?nmol (mg protein)^−2^. Figure adapted from [55].

However, recent observations by Li et al. [24] in a cell culture model are not in agreement with the above paradigm. When human cytosolic GPx-1 cDNA was transfected into a set of MnSOD-over-expressing U118 cells (a glioma cell line), they observed that:

- The GSSG content of these cells had a linear direct relation to the product of (GPx activity) × GSH, referred to as *effective GPx activity*. This is consistent with a higher rate of removal of H_2_O_2_ leading to an increase in GSSG;
- Intracellular ROS (oxidation within the cell), as measured by the change in fluorescence of intracellular dichlorofluorescin, had a linear inverse relationship to effective GPx activity. This is consistent with a higher steady-state level of H_2_O_2_ (Figure 2);
- The cell population doubling time had a linear inverse relationship to effective GPx activity, i.e. the greater the effective GPx activity, the faster the cells grew. This observation is coupled to the assumption that a higher effective GPx activity will lower the steady-state level of H_2_O_2_ and lead to a more reduced cellular redox environment and increased rate of growth [25]; and
- Most striking is that when the tumourigenicity of this set of cells with varying GPx activity was tested in nude mice, the growth rate of the tumours had a direct, linear relationship to effective GPx activity [24] (Figure 2). This is consistent with the *in vitro* observations, (a–c) above, and points to a fundamental role of H_2_O_2_ in setting the biological status of cells and tissues [5,25].

In the above study of Li et al. [24], over-expression of MnSOD and genetic modifications with respect to GPx-1 resulted in higher fluxes of H_2_O_2_ and various levels of GPx-1 in the cells. Because of the linear relationships with respect to [GPx][GSH] seen in Figure 2, these modifications appear not to have caused any significant changes in catalase or peroxiredoxin. Thus, the work of Li et al. serves as a reference for our modelling efforts to understand the GPx1-GSH-H_2_O_2_ system.

### Objective

The objective of this work is to examine the rate of removal of H_2_O_2_ with respect to the kinetic rate behaviour of GPx-1 and GSH. Justification of the kinetic model is possible by using the *in vivo* observations of Li et al. [24] to: (1) determine when the rate-results from the kinetic models are consistent with the observed effective GPx activity dependency; and (2) estimate the probable range of average cellular GPx and H_2_O_2_ in the cell lines investigated. To do this, we employed both the generalized and the classical approaches to express the kinetic rate behaviour involved in the GPx1-GSH-H_2_O_2_-system (equations 1–3) and extract concentration dependency from the overall system time constant, τ (also termed turnover time or biological ‘average life’ [26]). Finally, the variation of the classical model results from those of the general model was examined within this framework.

## Methods

### Generalized mathematical description of the removal of H_2_O_2_ by GPx

Often in determining the rate of removal of hydrogen peroxide, the concentration of GSH is assumed to be constant [27]. Invoking this approximation and assuming spatial independence, the transient behaviour of species described by equations (1–3) are a set of non-linear ordinary differential equations (ODEs) that describe the rates of change in the concentration of each species, equations (5–10). Here *C*_*i*_ represents the concentration of species *i*.

(5) $$\frac{dC_{GPx_{r}}}{dt}=k_{3}C_{GSH}C_{GSGPx}-k_{1}C_{GPx_{r}}C_{H_{2}O_{2}}$$

(6) $$\frac{dC_{H_{2}O_{2}}}{dt}=-k_{1}C_{GPx_{r}}C_{H_{2}O_{2}}$$

(7) $$\frac{dC_{GPx_{o}}}{dt}=k_{1}C_{GPx_{r}}C_{H_{2}O_{2}}-k_{2}C_{GSH}C_{GPx_{o}}$$

(8) $$\frac{dC_{H_{2}O}}{dt}=k_{1}C_{GPx_{r}}C_{H_{2}O_{2}}+k_{2}C_{GSH}C_{GPx_{o}}$$

(9) $$\frac{dC_{GSGPx}}{dt}=k_{2}C_{GSH}C_{GPx_{o}}-k_{3}C_{GSH}C_{GSGPx}$$

(10) $$\frac{dC_{GSSG}}{dt}=k_{3}C_{GSH}C_{GSGP_{x}}.$$

From a mathematical viewpoint, the experimental observations of Li et al. [24] can now be compared to the concentration dependency of the rate of removal of H_2_O_2_ for initial masses of H_2_O_2_, GPx and GSH introduced to the system (termed impulse response). These masses are described as equivalent initial concentrations. Since effective GPx activity proposed by Li et al. is the GPx activity coupled with GSH, we represent this as the product of initial GPx_r_ and GSH concentrations, [GPx_r_]_0_ × [GSH]_0_. This approximation is used to represent effective GPx activity for the purpose of investigating our kinetic rate models.

### Classical approximation of the rate of removal of H_2_O_2_ by GPx

Because of the inherent non-linearity of the generalized expressions for the rate of removal of H_2_O_2_,a traditional kinetic rate law approximation (the classical model) is typically used. The classical model, in fact, is derived from the generalized rate expressions. Using a steady-state approximation, assuming that the enzyme concentration is lower than the substrate concentration, the rate of change of all substrate-enzyme intermediates are negligible, the relationship between the initial rate, ν_0_, total enzyme concentration, *e*, and initial substrate concentrations, *S*_*i*_, for an enzymatic reaction with two substrates is approximated as [28]:

(11) $$\frac{e}{\nu_{0}}=\frac{\Phi_{1}}{\left[S_{1}\right]}+\frac{\Phi_{2}}{\left[S_{2}\right]}$$

where Φ_*i*_'s are functions of reaction rate constants, *k*_*i*_'s.

This approximation can be obtained from the general model (equations 5–10) by invoking several approximations for the kinetic rate model for the GPx1-GSH-H_2_O_2_ system. Starting with equations (5–10), by assuming constant concentrations of intermediates (equations 7 and 9, set to zero) and manipulating equation (6), one can obtain the classical rate expression for removal of H_2_O_2_, [16,29]:

(12) $$\frac{{\left[GPx_{r}\right]}_{0}}{-\frac{d[H_{2}O_{2}]}{dt}}=\frac{\Phi_{1}}{{\left[H_{2}O_{2}\right]}_{0}}+\frac{\Phi_{1}}{{\left[GSH\right]}_{0}},$$

where

(13) $$\Phi_{1}=\frac{1}{k_{1}}$$

And

(14) $$\Phi_{2}=\frac{1}{k_{2}}+\frac{1}{k_{3}}.$$

This classical expression results in a rate that is constant and depends only on the initial concentrations.

In this study, both the generalized and classical models are used to evaluate the rate of H_2_O_2_ removal. A comparison of relevant similarities and differences are provided.

### Parameters: Initial concentrations and reaction rate constants

In developing the model, we first need a range of concentrations that bracket expected physiological values. Using the data of Li et al. [24], we estimate the range of GSH in the five cell lines (Figure 2) to be 0.12–0.44 mM. Thus, we used the initial concentrations of 0.1–0.6 mM for GSH (Table II). However, there is no accurate way of correlating the data of Li et al. to GPx_r_ or H_2_O_2_ concentrations; their initial concentrations are estimated from related literature values.

**Table II:** Initial concentrations used for the GPx model.

Species	Initial concentration (m)
GSH	1 × 10^−4^, 2 × 10^−4^, 4 × 10^−4^, 6 × 10^−4^
GPx_r_	1 × 10^−7^, 5 × 10^−7^, 1 × 10^−6^, 5 × 10^−6^, 1 × 10^−5^, 5 × 10^−5^
H_2_O_2_	1 × 10^−7^, 5 × 10^−7^, 1 × 10^−6^, 5 × 10^−6^, 1 × 10^−5^, 5 × 10^−5^
GPx_o_	0
GS-GPx	0
GSSG	0

Most GPx is determined to be in its reduced form (>99%) from both *in vivo* studies [18] and mathematical simulations [27]. Therefore, we assumed all GPx in our model to be initially in the reduced form, GPx_r_. Estimated cellular concentrations of GPx vary from 0.2 μM in red blood cells [18] to values of 2.5 μM and 6.7 μM derived from mathematical models [27,30]. Rat liver cytosolic GPx-1 has been estimated to be 5.8 μM from Se of 0.46 ppm [31]; total GPx (monomer) in mitochondria and in the luminal space of endoplasmic reticulum is estimated to be 10 μM and 0.32 μM, respectively [32]. These values may be an over-estimate as we now know that there are additional Se-containing enzymes, e.g. thioredoxin reductase [33]. As suggested by the vast difference in reported concentrations, our initial GPx_r_ used in our modelling ranges from 0.1–50 μM (Table II).

The concentration of H_2_O_2_ in organisms can vary widely, from 0.2 nM in red blood cells to as high as 200 μM in wound fluid [34,35]. Concentrations of H_2_O_2_ in rat liver cells have been found to range from 10^−9^−10^−7^ M [36]. A recent survey of intracellular [H_2_O_2_] has estimated 700 nM in non-patholo-gical conditions [37]. This upper limit of 700 nM is suggested because intracellular levels above this value induce apoptosis in Jurkat T-cells [38]. Reportedly, H_2_O_2_ was found to be able to reach 7 μM in cytosol and 2 μM in mitochondria [39]. To capture the higher level of H_2_O_2_ due to the over-expression of MnSOD in the genetically-modified glioma cells used by Li et al. [24], the range of initial H_2_O_2_ chosen for our model was varied from 0.1–50 μM (Table II).

Rate constants for equations (1–3) have been determined in dilute buffer solutions [16,18,23]. These rate constants vary depending on conditions such as the buffer-salt and pH of the solution. Rate constants used (Table I) represent estimates of the effective intracellular rate constants for the three principal steps of the GPx catalytic cycle [30].

### Time constant for the removal of H_2_O_2_

In order to search for ranges of possible physiological GPx_r_ and H_2_O_2_ for cell lines under conditions used by Li et al. [24], time-dependent numerical solutions given by our model of the GPx1-GSH-H_2_O_2_ system are correlated to the observations of Li et al. As shown in Figure 2, the data of Li et al. present a linear relation between the effective GPx activity and the relative cellular H_2_O_2_. This biological observation can be compared to the concentration dependency of the rate of removal of H_2_O_2_. The dependency is generally reflected in an analytical solution for the overall system time constant, τ (turnover time), provided that the model is linear. The overall rate by which the system evolves is dominated by this approximated time constant in the system. Thus, the functional dependency of twill allow us to understand the kinetic behaviour of the GPx1-GSH-H_2_O_2_ system.

However, because of the non-linearity of the rate equations associated with the removal of H_2_O_2_ (due to the coupling of time-dependent concentrations of species in the terms on the right-hand side of each expression (equations 5–10), a closed-form solution does not exist. For non-linear systems, τ can be approximated.

### Relating overall system time constant to effective GPx activity

To meet our objectives, we have determined the dependency of effective GPx activity on τ for the chosen range of initial GSH, GPx_r_ and H_2_O_2_ concentrations. Specifically, this is when τ is inversely proportional to effective GPx activity, consistent with the observations of Li et al. [24],

(16) $$\tau \infty \frac{1}{C_{GSH}C_{GPx}}.$$

Then, comparing these values to acceptable physiological conditions for the genetically-modified cells used by Li et al. [24], we will pose possible ranges of average cellular GPx and H_2_O_2_.

The initial conditions for variables held constant are shown in Table II. There are six initial concentrations used for H_2_O_2_,[H_2_O_2_]_0_, in our models. For every [H_2_O_2_]_0_, there are six different initial concentrations used for GPx_r_, [GPx_r_]_0_. Similarly, for each [GPx_r_]_0_ there are four initial concentrations used for GSH, [GSH]_0_. This results in 144 cases for each general and classical model.

The time constant, τ, of interest here is the time taken for a 63% decay in H_2_O_2_. For the general model, τ for removal of H_2_O_2_ can be extracted from the numerical solutions of the generalized rate expressions (equations 5–10). Since the rate of removal of H_2_O_2_ given by the classical approach is independent of time, τ can be directly calculated by integrating equation (12).

### Numerical methods

All equation-sets were solved with initial concentrations and rate constants, listed in Tables I and II. Species rate expressions, shown in equation (5–10), are therefore numerically integrated by using the IMSL (International Mathematical and Statistical Library) DIVPAG (double-precision initial value problem solver using either Adam-Moulton's or Gear's method) coded using Fortran [40–42].

## Results and discussion

### Mathematical ranges of concentrations demonstrating effective GPx activity dependency

**Figure 3:**
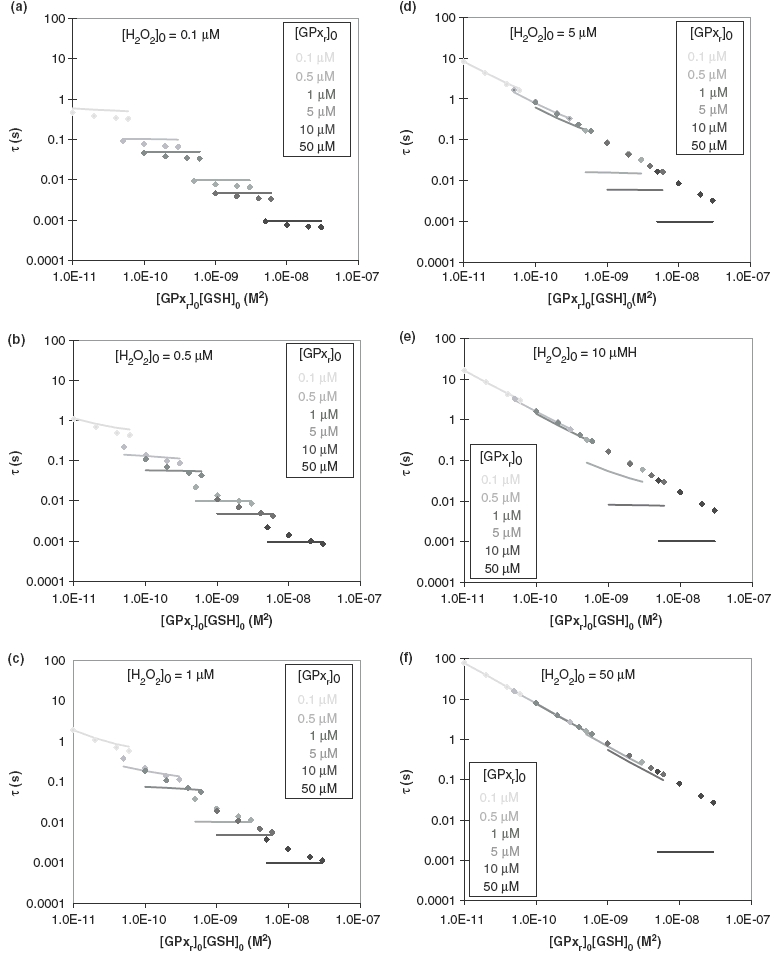
Model results in determining linear dependency of time constant with respect to effective GPx activity. The dependence of the time constant, τ, on effective GPx activity, approximated by [GPx_r_]_0_[GSH]_0_, are shown on log-log plots for various initial concentrations of GPx_r_ and H_2_O_2_. Results from the general model are shown as solid lines; results from the classical model are shown as dotted lines. The short segments result from calculations of effective GPx activity for a fixed [GPx_r_]_0_ with a span on [GSH]_0_ (0.1, 0.2, 0.4 and 0.6 mM). If the line segments are parallel to the abscissa, then there is no dependence of τ on [GSH] in the range of concentrations tested; if the line-segments show a non-zero slope, then there is dependence of [GSH]. The system would be completely dependent on effective GPx activity if all points fell on a single straight line. [GPx_r_]_0_ used for both models are 0.1 μM, 0.5 μM, 1 μM, 5 μM, 10 μM and 50 μM. (a) [H_2_O_2_]_0_ = 0.1 μM; (b) [H_2_O_2_]_0_ = 0.5 μM; (c) [H_2_O_2_]_0_ = 1 μM; (d) [H_2_O_2_]_0_ = 5 μM; (e) [H_2_O_2_]_0_ = 10 μM; (f) [H_2_O_2_]_0_ = 50 μM. The general model captures dependency when [H_2_O_2_]_0_ is 5 μM and [GPx_r_]_0_ is ≤1 μM. Note that the classical model under-predicts the [H_2_O_2_]_0_ for the onset of effective GPx activity dependency. Furthermore, the estimated time constants for the classical model can be orders of magnitude different than that determined from the more general kinetic model.

In Figure 3 are plotted values of all τ obtained from both the general and classical models, organized for each [H_2_O_2_]_0_, v the values of [GPx_r_]_0_[GSH]_0_ on a log-log scale. Time constants from the general model for [H_2_O_2_]_0_ of 0.1–50 μM are shown as solid lines in Figure 3(A–F). In each figure panel, corresponding to a given [H_2_O_2_]_0_, [GPx_r_]_0_ ranges from 0.1–50 μM, shown with various colours. For each [GPx_r_]_0_ there are four [GSH]_0_ (0.1, 0.2, 0.4 and 0.6 mm) that make up each line. In Figure 3(a), the reaction starts with [H_2_O_2_]_0_ of 0.1 μM. As expected, for cases where [GPx_r_]_0_ > [H_2_O_2_]_0_, there is no GSH dependency; tis inversely proportional to [GPx_r_]_0_ only. When the system starts with equal amounts of [GPx_r_]_0_ and [H_2_O_2_]_0_, τ begins to show both GPx_r_-dependency and slight GSH-dependency for cases with lower [GSH]_0_. Similar trends are observed as [H_2_O_2_]_0_ increases, as seen in Figure 3(b–f).

There is little or no GSH-dependency on τ when [GPx_r_]_0_ > [H_2_O_2_]_0_. This clearly shows that the rate of removal of H_2_O_2_ is not a function of [GSH]_0_. In these cases, τ is inversely proportional to [GPx_r_]_0_; therefore, the system's ability to remove H_2_O_2_ is not affected by the recycling of GPx_r_ or the amount of GSH available.

Only when [GPx_r_]_0_ < [H_2_O_2_]_0_ does τ begin to show dependency on both [GPx_r_] and [GSH], i.e. the time needed for removal of H_2_O_2_ increases and is clearly a function of both [GPx_r_]_0_ and [GSH]_0_. The removal of H_2_O_2_ in these cases depends on the continuous recycling of GPx_r_ and the amount of GSH available to recycle GPx_r_ becomes important. These results are in agreement with the analysis of Flohe´ and colleagues [16,18,19]. It is generally believed that [GPx_r_] > [H_2_O_2_] in cells and tissues. However, both the observations of Li et al. [24] and our kinetic model imply that these conditions are not always true.

Based on our generalized mathematical model, there exist sets of initial GPx_r_ and GSH concentrations within all ranges studied where τ is generally inversely proportional to [GPx_r_]_0_[GSH]_0_ for the removal of H_2_O_2_, agreeing with the findings of Li et al. [24] shown in Figure 2 and the relationship expressed in Equation (16). This linear relationship between τ and [GPx_r_]_0_[GSH]_0_ is clearly visible for the following cases:

1. When [H_2_O_2_]_0_ is 5, 10 and 50 μM, as shown in Figure 3(d–f), for [GPx_r_]_0_ of 0.1, 0.5 and 1 μM; and,
2. When [H_2_O_2_]_0_ is 50 μM, as shown in Figure 3(F), for [GPx_r_]_0_ of 0.1, 0.5, 1, 5 and 10 μM.

### Implications of modelling results relative to the observed biological phenomena

Mathematical modelling demonstrates that the rate of removal of H_2_O_2_ can be a function of [GPx_r_]_0_[GSH]_0_, specifically when [GPx_r_]_0_ < [H_2_O_2_]_0_ and the recycling of GPx_r_ is rate-limiting. Assuming the rate of production of H_2_O_2_ is on the same order as the rate of removal, varying [GPx_r_]_0_[GSH]_0_ would change the steady-state level of H_2_O_2_. This is consistent with Li et al.'s [24] observations. When the U118 cells of Li et al. were genetically manipulated to change [GPx_r_]_0_[GSH]_0_, the apparent steady-state level of H_2_O_2_ varied inversely with [GPx_r_]_0_[GSH]_0_. The in vivo observations presented in Figure 2 clearly demonstrate that effective GPx activity ([GPx_r_]_0_ [GSH]_0_) correlates with biochemical and biological properties. Most striking is that this is associated with the rate of growth for tumours. Thus, effective GPx activity appears to be an important biochemical parameter to monitor and use to understand the biology associated with differing fluxes of H_2_O_2_ and the role of the peroxide-removing system.

Although the rate of elimination of H_2_O_2_ *in vivo* by GPx is generally assumed to be independent of [GSH], the results of the kinetic simulation indicated that the rate of peroxide-removal can potentially be a function of [GSH]. To help explain this we have to address the range of initial concentrations ([GPx]_0_, [GSH]_0_,[H_2_O_2_]_0_) used, which are estimated from the U118 cells of Li et al. [24]. Reported levels of GSH and activities of GPx of other cells are compared with those of the U118 cells.

Typical levels of GSH in cells range from 1–10 mm [25]. From the data of Li et al. [24] on the level of GSH in U118 cells and a cellular volume of 2.4 pL (F.Q. Schafer, unpublished), we estimated the range of GSH in the five cell lines of Figure 2 to be 0.12–0.44 mm. This is 10-times smaller than concentrations typically observed in proliferating cells.

The measured activity of GPx in the set of cells studied ranged from 15–65 mU/mg protein (using the assay and unit definition of [43]). [GPx] is considered to be at lower levels in tumour cells and cancer [6,44–48]. These values are comparable to the range of values published for other cancer cell lines, e.g. PC-3 cells, 18 mU/mg protein [49]; MCF7, 38 mU/mg protein; MDA-MB231, 98 mU/mg protein; and MCF-10A, 218 mU/mg protein [50]. These comparisons point to the low levels of GSH in U-118 cells as being a contributor to Li et al.'s [24] observation that peroxide levels and tumour growth are a function of (GPx activity) × [GSH].

The time constant results provided by the general model indicate that if the possible intracellular concentration of H_2_O_2_ is in the range of 5–50 μM, then the physiological concentration of GPx is likely to be between 0.110 μM. However, as mentioned above, the upper limit for intracellular [H_2_O_2_]in normal cells is proposed to be ∼ 700 nm [37,38]. However, the genetically-modified glioma cells used by Li et al. [24] over-expressed MnSOD by as much as 5-fold. This increase in MnSOD will likely increase the steady-state concentration of H_2_O_2_ [1]. Therefore, a predicted physiological range of [H_2_O_2_]and [GPx_r_] for the results of Li et al. are approximately [GPx_r_] ≤ 1 μM and [H_2_O_2_] ∼ 5 μM.

It should be noted that actual concentrations may vary from those proposed by our model. This is because the modelling results are a consequence of the selected reaction rate constants and initial concentrations used in Equations (1–3).

Finally, it is important to recognize that, in our modelling of the removal of H_2_O_2_ by the GPx-GSH-H_2_O_2_ system, spatially dependent concentrations were not considered and cellular averages were used. However, gradients in the intracellular concentrations clearly exist [6,37,51] and can result in local dominance of the rate of removal of H_2_O_2_ that can alter our predicted cellular average concentrations.

### Deviations of the classical model from the general model results

Time constants obtained from the classical model for [H_2_O_2_]_0_ in the range of 0.1–50 μM are also shown in Figure 3(a–f) as dots representing all cases studied. Unlike the general model, τ shows dependency on both [GPx_r_] and [GSH] for the entire range of concentrations tested. Linear dependency of τ on [GPx_r_]_0_[GSH]_0_ can be observed when [H_2_O_2_]_0_ varies from 1–50 μM for [GPx_r_]_0_ of 0.1–50 μM (Figure 3(c–f)). Although the resulting values for τ from the classical model deviate noticeably from the general model for most cases, they agree within the probable physiological ranges of [GPx_r_] and [H_2_O_2_] suggested by the general model. This behaviour occurs as a result of the assumptions in the classical model that the enzyme concentration is lower than that of the substrate. Therefore, under this condition of relatively low [GPx_r_] and high [H_2_O_2_], both models should agree well, especially for the low [GSH] found for U118 cells.

**Figure 4:**
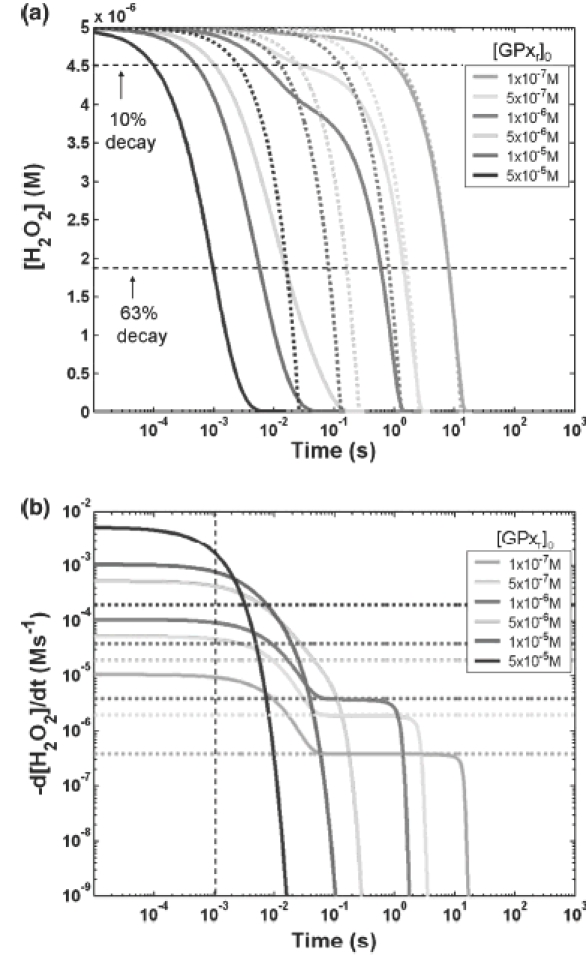
Example cases demonstrating deviations between the generalized and classical model results. Cases used here, as examples to demonstrate discrepancies, are for initial concentration of H_2_O_2_, [H_2_O_2_]_0_, of 5 μM and initial concentration of GSH, [GSH]_0_, of 0.1 mm. (A) Transient H_2_O_2_ profiles for various [GPx_r_]_0_ of the general (solid lines) and classical (dotted lines) models are shown in this semi-log plot. Even for cases with lower [GPx_r_]_0_, where the time needed for 63% decay (time constants, τ) from both models agree well, the classical model is not a good approximation throughout. For example, in the case where [GPx_r_]_0_ is 1 μM (magenta), the time taken for 10% decay given by both models are a factor of 10 different. (b) The rates of disappearance of H_2_O_2_ for various [GPx_r_]_0_ of the general (solid lines) and classical (dotted lines) models are plotted with a semi-log scale. Using the same case of where [GPx_r_]_0_ is 1 μM (magenta), at physiological turnover time for H_2_O_2_ of ms, the rate of removal of H_2_O_2_ given by the general model is approximately a factor of 100 greater than the classical rate.

However, due to simplifications made in deriving the classical rate expression, the classical model is less sensitive in capturing the full behaviour of the removal of cellular H_2_O_2_. Using the case where [H_2_O_2_]_0_ is 5 μM and [GSH]_0_ is 0.1 mm, transient [H_2_O_2_] profiles for both the general (solid lines) and classical (dotted lines) models are presented on a semi-log plot (Figure 4(a)). The [H_2_O_2_] from the classical model is calculated by integrating the rate expression shown in Equation (12). The time taken for 63% decay (which is ∼ τ) in both models agrees relatively well for the three cases where [GPx_r_]_0_ is 0.1, 0.5 and 1 μM (as also shown in Figure 3(d)). For example, in the case where [GPx_r_]_0_ is 1 μM, although τ's given for both models are close, the times predicted for 10% decay by the two models are more than an order of magnitude different. The rates of removal of H_2_O_2_ at 1 ms given by the two models, as shown in Figure 4(B), are two orders of magnitude different. These differences, which occur early during reactions, could result in substantial cumulative discrepancies.

**Figure 5:**
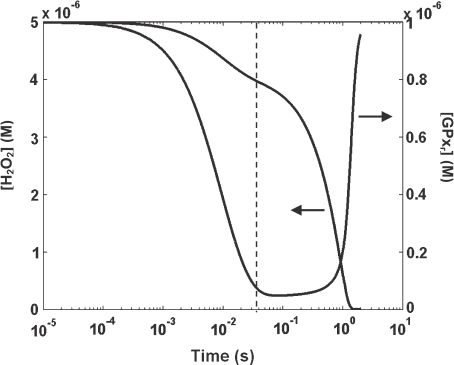
Example of the rate-limiting effect of the slower GPx_r_ recycling step. Concentration profiles of H_2_O_2_ and GPx_r_ of the general model are shown for [H_2_O_2_]_0_ of 5 μM, [GSH]_0_ of 0.1 mM and [GPx_r_]_0_ of 1 μM. The inflection point on the [H_2_O_2_]-profile that occurs around 40 ms corresponds to the change in the rate of production of GPx_r_.

Furthermore, for the same case, the H_2_O_2_ profile given by the classical model does not capture the inflection point where there is an obvious change in the rate of removal of H_2_O_2_. The slower rate of H_2_O_2_ removal is due to the slow recycling of GPx_r_ as shown in a plot comparing the transient [H_2_O_2_] and [GPx_r_] (Figure 5). These points of inflection are clearly visible for cases where [GPx_r_]_0_ < [H_2_O_2_]_0_, (Figure 4(a)). When [H_2_O_2_] is initially higher than [GPx_r_], then [GPx_r_] is the rate-limiting factor. When this is the case, the continuation of the H_2_O_2_-eliminating reaction of Equation (1) depends on the amount of GPx_r_ being recycled. This is particularly true when the recycling reaction steps, shown in equations (2) and (3), are much slower compared to the H_2_O_2_ eliminating step. The reaction rate constant for Equation (2) is three orders of magnitude smaller than the rate constant for equation (1); the rate constant for Equation (3) is very near that of equation (1). Thus, Equation (2) would be a rate-limiting reaction in the recycling of GPx_r_. In cases with lower [GPx_r_] and [GSH], the slow recycling effect becomes more significant at earlier times during the process.

Nevertheless, these discrepancies are based on the set of initial concentrations used, as illustrated in Figure 4(B). Using the same example where [H_2_O_2_]_0_ is 5 μM and [GSH]_0_ is 0.1 μM, for cases with [GPx_r_]_0_ of 0.1, 0.5 and 1 μM, there exists a steady-state region for the rate of removal of H_2_O_2_ given by the general model. This steady-state rate is concurrent to steady-state [GPx_o_] and [GS-GPx]. Since the classical rate expression is derived by invoking the steady-state approximation on GPx_o_ and GS-GPx, the rate given by the classical model should be in agreement with this steady-state rate given by the general model, as seen in Figure 4(b).

Finally, modelling the removal of H_2_O_2_ by the GPx-GSH-H_2_O_2_ system is a multi-scale problem and is spatially dependent. The time scale for removal of H_2_O_2_ is on the order of milliseconds [27,52] whereas cell growth is on the order of days. Therefore, small differences in modelling solutions could significantly impact long-term predicted behaviour. For this reason, the classical approach to expressing the rate of enzymatic reactions should be used with caution, especially when addressing more complex systems.

## Conclusions

With the use of kinetic modelling, we have investigated the removal of H_2_O_2_ by GPx. Our goal was to examine the concentration dependency of intracellular H_2_O_2_ removal to understand the anomalies in the findings of Li et al. [24]. They observed that biochemical parameters related to the removal of H_2_O_2_ in genetically-modified U118-9 cells were a function of effective GPx-activity; most striking was their observation that the rate of tumour growth in an animal model was directly related to effective GPx activity. Using mathematical modelling, with sets of reaction rate constants and initial species concentrations taken from the literature, we found that:

- as expected, the rate of removal of H_2_O_2_ increased with [GPx_r_]_0_;
- the rate of removal of H_2_O_2_ is affected by [GPx_r_]_0_ and [GSH]_0_ when [GPx_r_]_0_<[H_2_O_2_]_0_; the reason for this is the slow recycling of GPx_r_;
- the overall time constant, τ, is inversely proportional to the product [GPx_r_]_0_ × [GSH]_0_, as shown in equation (16); this holds for intracellular concentrations of GPx_r_ ≤ 1 μM with [H_2_O_2_]≥ 5 μM and for GPx_r_ ≤ 10 μM with [H_2_O_2_] ≥ 50 μM;
- the plausible concentrations for U118 cells of Li et al. [24] are predicted to be ∼ GPx_r_ ≤ 1 mmand [H_2_O_2_] ∼ 5 μM;
- the classical approach to deriving the rate of removal of H_2_O_2_, as expressed in equation (12), matches the generalized rate favourably when species concentrations corresponding to steady-state [GPx_o_] and [GS-GPx] are used;
- but, while offering useful simplicity, under certain conditions, the classical approach can result in substantial differences from the more general form over long time periods.

In the future, to further examine this system, the current lumped parameter mathematical model should be refined to include spatial dependency and H_2_O_2_ generation. Issues of transport properties, such as species diffusivities and membrane permeability, and reaction rate constants, perhaps due to the crowded environment [53,54], need to be investigated. A direct coupling of cell growth to H_2_O_2_ residence time is required to connect mathematical simulation to biological observations.

Mathematical modelling made it possible to quantitatively study the time constants (turnover time) associated with the removal of H_2_O_2_ by GPx, providing insight into a biological observation that could not be approached experimentally. Finally, modelling demonstrates that the paradigm established from the kinetic-observations in dilute aqueous buffer do not always hold in the complex milieu of the cell.

## Abbreviations

*C*_j_: concentration of species *j*
$\frac{dC_{j}}{dt}$: rate of change of species *j*
DCFH_2_: 2′,7′-dichlorodihydrofluorescein
GPx: glutathione peroxidase
GPx-1: classic (cytosolic) glutathione peroxidase
GPx_o_: oxidized glutathione peroxidase
GPx_r_: reduced glutathione peroxidase, the form that reacts with hydroperoxides
GS-GPx: glutathione-enzyme complex
GSH: glutathione
GSSG: glutathione disulphide
*k*_*i*_: reaction rate constant of reaction *i*
ODE: ordinary differential equation
ROS: reactive oxygen species
τ: overall time constant
[*i*]_0_: initial concentration of species *i*
